# Phase delay and neural dyssynchrony of the 40-Hz auditory steady-state response in mood and psychotic disorders

**DOI:** 10.3389/fpsyt.2026.1879294

**Published:** 2026-09-01

**Authors:** Masaya Yanagi, Shizuka Ishida, Osamu Shirakawa, Mamoru Hashimoto

**Affiliations:** Department of Neuropsychiatry, Faculty of Medicine, Kindai University, Osaka, Japan

**Keywords:** 40-Hz ASSR, bipolar disorder, major depressive disorder, phase delay, phase locking, schizophrenia

## Abstract

**Background:**

Impaired gamma-band neural synchrony has emerged as a promising biomarker for precision psychiatry; however, clinically scalable methods for individual-level assessment remain limited. Widely available evoked response audiometry (ERA) systems offer a pragmatic and accessible platform for measuring the 40-Hz auditory steady-state response (ASSR), and their utility has been demonstrated in patients with schizophrenia. This study investigated whether ERA-based profiling could be extended to mood disorders, which are believed to share underlying biological mechanisms with schizophrenia.

**Methods:**

Using a conventional ERA system, phase delay and phase-locking status of the 40-Hz ASSR were assessed in 107 participants, including individuals with schizophrenia (n = 29), bipolar disorder (n = 24), major depressive disorder (n = 25), and healthy controls (n = 29). Phase locking status was classified as either a phase-locked response or a nonphase-locked response (NPLR).

**Results:**

Compared with the healthy control group, all clinical groups exhibited significant phase delays in the 40-Hz ASSR. Phase-locking analysis identified NPLR not only in individuals with schizophrenia but also in a subset of participants with bipolar disorder and major depressive disorder.

**Conclusion:**

These findings extend previous evidence of 40-Hz ASSR phase delay and demonstrate that individual-level NPLR assessment using a conventional ERA system applies beyond schizophrenia to bipolar disorder and major depressive disorder. ERA-based evaluation of the 40-Hz ASSR may represent a practical and scalable approach for biomarker development in mood and psychotic disorders.

## 1 Introduction

Converging evidence from genetic and neuroanatomical studies indicates substantial biological overlap among schizophrenia, bipolar disorder, and major depressive disorder (1–4). These findings challenge traditional categorical boundaries and prompted the development of dimensional, biologically informed frameworks for precision psychiatry (5–7). Despite this paradigm shift, few state-sensitive biomarkers have proven sufficiently robust and scalable for routine clinical use while reliably linking neural dysfunction to clinically relevant dimensions (8–10).

Gamma-band oscillations reflect inhibitory–excitatory synchrony within cortical networks (11, 12), and the 40-Hz auditory steady-state response (ASSR) is a well-established electrophysiological measure of gamma oscillatory function. Consistent reductions in 40-Hz ASSR phase locking have been reported in schizophrenia (13, 14). Similar abnormalities have also been observed in bipolar disorder (15, 16), although the impairments generally appear less pronounced than those reported in schizophrenia (17, 18). Findings in major depressive disorder have been less consistent. While some studies have reported reduced phase locking of the 40-Hz ASSR (19, 20), others have found no significant impairment (21, 22). These discrepancies may partly reflect the limitations of group-level averaging, which can obscure biologically homogeneous subgroups, particularly when such subgroups represent only a small proportion of the diagnostic population.

Currently, most psychiatric studies of the 40-Hz ASSR lack validated criteria for identifying abnormalities at the individual level. Evoked response audiometry (ERA) systems, which are routinely used in otolaryngology, provide a practical and scalable platform for individual-level assessment. These systems include predefined thresholds for evaluating 40-Hz ASSR, allowing responses to be classified as either normal or impaired. Although ERA systems are limited by single-channel recordings and a relatively restricted range of analytical parameters, they offer considerable advantages in terms of rapid and objective classification of ASSR responses. A recent study has shown that ERA-based 40-Hz ASSR assessment can identify a subgroup of individuals with schizophrenia who exhibit nonphase-locked responses (NPLR), representing a neural dyssynchrony phenotype, whereas no healthy controls demonstrated this abnormal response pattern (23). However, it remains unknown whether ERA-based individual-level profiling can identify NPLR beyond schizophrenia, particularly in bipolar disorder and major depressive disorder.

In addition to impaired phase locking, the delayed phase of the 40-Hz ASSR has emerged as an independent and potentially more sensitive neurophysiological marker of schizophrenia (24). Whereas reduced phase locking reflects an inability of cortical networks to synchronize with auditory stimulation, phase delay reflects delayed neural timing despite preserved synchronization. To our knowledge, no previous study has investigated whether this electrophysiological abnormality extends across mood disorders. Therefore, the present study aimed to establish a scalable framework for evaluating the 40-Hz ASSR using a conventional ERA system by characterizing phase delay across schizophrenia, bipolar disorder, and major depressive disorder while simultaneously identifying individual-level neural dyssynchrony through classification of phase-locked responses (PLR) and NPLR. Furthermore, we explored the relationships between neural synchrony profiles and dimensional clinical characteristics across traditional diagnostic boundaries.

## 2 Methods

### 2.1 Participants

This study analyzed data from 107 participants, including 25 individuals with major depressive disorder and 24 individuals with bipolar disorder who were newly recruited for the present study, as well as 29 individuals with schizophrenia and 29 healthy controls drawn from a previously reported cohort (23). The same psychiatrists at Kindai University who recruited participants with schizophrenia also recruited those with mood disorders. Clinical information was obtained through longitudinal outpatient follow-up and detailed inpatient assessments conducted by the treating psychiatrists. Diagnoses were independently confirmed by two experienced research psychiatrists according to the DSM-5. Eligible participants had a diagnosis of schizophrenia, bipolar disorder, or major depressive disorder without comorbid psychiatric disorders. Individuals experiencing manic episodes or acute psychotic states at the time of recruitment were excluded. Most participants were clinically stable, with no major changes in psychotropic medication during the month preceding enrollment. Psychiatric symptoms and overall functioning were evaluated using the Brief Psychiatric Rating Scale (BPRS) (25) and the Global Assessment of Functioning (26), respectively. BPRS scores were further categorized according to the four-factor model (27): thought disturbance (hallucinatory behavior, unusual thought content, conceptual disorganization), withdrawal–retardation (emotional withdrawal, blunted affect, and motor retardation), hostility–suspiciousness (hostility, suspiciousness, and uncooperativeness), and anxious depression (anxiety, guilt, and depressive mood). The thought disturbance and withdrawal–retardation factors broadly correspond to positive and negative symptom dimensions, respectively (28). Demographic and clinical characteristics are summarized in **Table 1**. Healthy controls were recruited from hospital staff and underwent screening interviews conducted by psychiatrists to exclude any history of psychiatric, neurological, or hearing disorders. All participants provided written informed consent before enrollment. This study was approved by the Ethics Committee of Kindai University Faculty of Medicine and was conducted in accordance with the Declaration of Helsinki and its amendments.

**Table 1:** Clinical variables of the study participants across diagnostic groups.

	Schizophrenia		Bipolar disorder		Major depressive		Healthy controls		Statistics		
	(n = 29)		(n = 24)		Disorder	(n = 25)	(n = 29)				
	Mean	SD	Mean	SD	Mean	SD	Mean	SD	Statistic	df	p
Age (years)	52.2	9.4	53.8	11.3	53.0	17.2	52.4	8.2	*F* = 0.1	df = 3, 103	*p* = 0.97
Age at onset (years)	24.8	9.1	37.2	10.1	44.5	17.9	–	–	*F* = 16.5	df = 2, 75	*p* < 0.0001
Duration of illness (years)	27.5	11.1	16.5	9.5	8.4	7.8	–	–	*F* = 26.5	df = 2, 75	*p* < 0.0001
BPRS, total scores	23.9	11.8	8.4	7.2	11.4	11.3	–	–	*F* = 16.9	df = 2, 75	*p* < 0.0001
BPRS, four-dimensional model											
Thinking disturbance	6.7	4.2	1.0	1.3	0.9	1.8	–	–	*F* = 37.2	df = 2, 75	*p* < 0.0001
Withdrawal-retardation	5.3	4.1	1.6	2.1	2.8	3.7	–	–	*F* = 8.1	df = 2, 75	*p* = 0.0007
Hostile-suspiciousness	2.8	2.8	1.1	2.1	1.0	1.8	–	–	*F* = 5.3	df = 2, 75	*p* = 0.007
Anxious depression	2.8	2.2	2.1	1.9	4.1	2.8	–	–	*F* = 4.6	df = 2, 75	*p* = 0.01
Global assessment of functioning score	32.8	16.6	61.0	24.3	57.9	23.9	–	–	*F* = 14.0	df = 2, 75	*p* < 0.0001
Antidepressants^a^ (mg/day)	19.8	55.7	52.6	82.9	146.6	140.5	–	–	*F* = 11.8	df = 2, 75	*p* < 0.0001
Lithium (mg/day)	69.0	181.5	504.2	282.0	56.0	196.0	–	–	*F* = 33.0	df = 2, 75	*p* < 0.0001
Antipsychotics^b^ (mg/day)	1024.0	1171.0	181.9	232.2	109.3	151.7	–	–	*F* = 13.2	df = 2, 75	*p* < 0.0001
Benzodiazepines^c^ (mg/day)	8.1	7.6	7.8	12.0	5.8	9.8	–	–	*F* = 0.42	df = 2, 75	*p* = 0.66
Sex, female/male (*n*)	16/13		9/15		14/11		14/15		*χ*^2^ = 2.2	df = 3	*p* = 0.53

^a^ Imipramine equivalent dose.

^b^ Chlorpromazine equivalent dose.

^c^ Diazepam equivalent dose.

### 2.2 ASSR recording

Participants were seated comfortably in a quiet environment and instructed to relax, keep their eyes closed, avoid falling asleep, and remain as still as possible throughout the recording to minimize muscle artifacts. ASSR recordings were obtained using the commercially available ERA system Audera (Grason-Stadler Inc., MN, USA), in accordance with a custom protocol incorporating minor modifications, as previously described (23). Auditory stimuli were delivered binaurally through insert earphones by replacing the standard single tube with a bifurcated tube. The stimuli consisted of a continuous 1000-Hz carrier tone presented at 70 dB and amplitude-modulated at 40 Hz with a modulation depth of 100%, thereby generating continuous 40 Hz amplitude fluctuations without interstimulus intervals. The standard Audera recording protocol specifies the recording and ground electrodes as being placed on the high forehead and low forehead, respectively. To ensure consistent electrode placement across participants, the recording and ground electrodes were positioned at AFz and Fpz, respectively, according to the international 10–10 electroencephalographic (EEG) system. The reference electrode was positioned on the left earlobe. EEG activity was recorded using disposable self-adhesive electrodes (Neuroline 720 Ambu, Denmark). Electrode impedance was maintained below 5 kΩ through appropriate skin preparation using Nuprep conducive gel (Weaver and Company, CO, USA).

### 2.3 ASSR analysis

**Figure 1:**
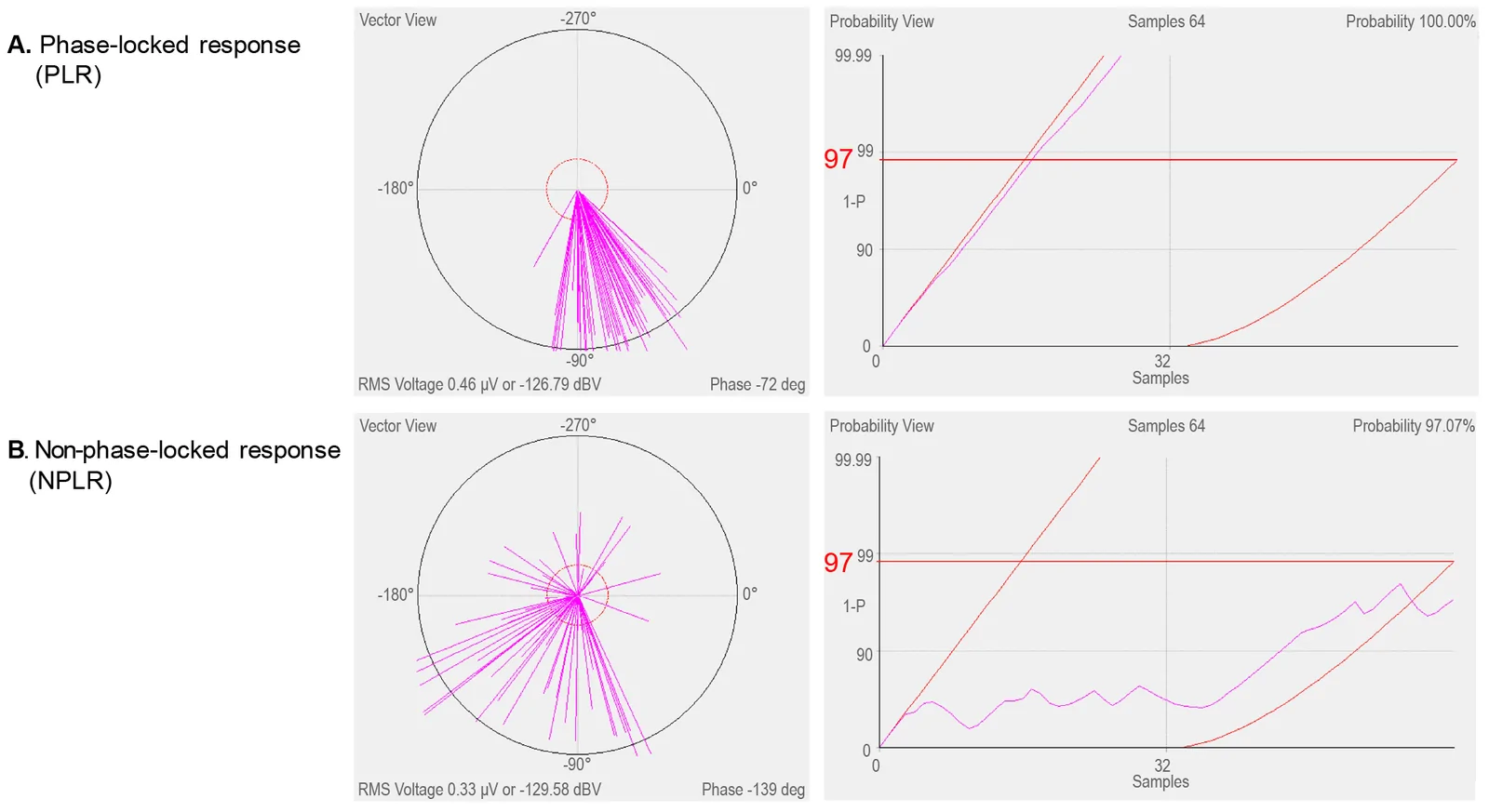
Individual-level classification of 40-Hz ASSR using a conventional ERA system. The ERA platform provides statistical phase-coherence thresholds, which were used in this study to classify individual responses as either **(A)** phase-locked responses or **(B)** nonphase-locked responses. In the left panels, each pink vector represents the ASSR amplitude (root mean square voltage, μV) and phase (degree) for an analytical sample. The inner red and outer black circles denote amplitudes of 0.1 and 0.5 μV, respectively. Phase represents the temporal lag between auditory stimulation and the neural response and is displayed as a negative angle relative to 0°. ASSR recordings consisted of 64 analytical samples per participant (the device-defined maximum). Because stimulation was continuous and did not include interstimulus intervals, recording time remained relatively short (approximately 98 s for 64 sample lengths), helping to reduce the likelihood of participants falling asleep during the assessment. Mean phase values calculated across these 64 samples were used for subsequent between-group analyses. The right panels illustrate the statistical evaluation of phase locking. As analytical samples accumulate, probability values are recalculated and displayed as (1 − *p*), shown by the pink line. The horizontal red line indicates the device’s predefined ASSR detection threshold (97%; *p* = 0.03). Responses exceeding this threshold were classified as PLRs (upper panel), whereas responses not exceeding the threshold were classified as NPLRs (lower panel). This framework provides an immediately interpretable measure of individual-level neural synchrony.

ASSR analysis was performed automatically using statistical algorithms implemented in the Audera system, which are based on the established objective detection methods employing frequency-domain and phase-based analyses (29, 30). Following the system’s default analog filtering (0.2–10,000 Hz, the continuous EEG signal was segmented into consecutive epochs. Every 10 consecutive epochs are averaged to generate a single analytical sample for ASSR analysis. Based on a previous evaluation of the Audera system, each analytical sample corresponds to approximately 1.5 s of recording (31). Fast Fourier transformation was applied to each analytical sample to determine the amplitude (root mean square voltage, µV) and phase (degree) at the 40-Hz modulation frequency. Each sample was represented as a vector defined by its amplitude and phase (**Figure 1**, left panels). As additional analytical samples accumulated during the recording, the number of vectors increased, and the mean amplitude and phase were updated continuously in real time. To ensure reliable estimation of phase, recordings were continued until the device-defined maximum of 64 analytical samples was reached, corresponding to approximately 98 s of recording, including the initial acclimation period. Phase-locking status was determined using the system’s built-in statistical algorithm. Squared phase coherence (PC²) was calculated to quantify phase consistency across analytical samples, and statistical significance was evaluated using circular variance. The device applies a predefined confidence threshold of 97% ASSR detection. Responses exceeding this threshold were classified as PLR, whereas those failing to reach the threshold were classified as NPLR (**Figure 1**, right panels). For all subsequent analyses, phase-locking status and mean phase values derived from the 64 analytical samples were used.

### 2.4 Statistics

Demographic and clinical variables were compared using analysis of variance or chi-square tests as appropriate. Between-group differences in Audera-derived 40-Hz ASSR phase were evaluated using the Watson–Williams test, a circular statistical method for comparing mean angles between groups, as described previously (24, 32). The frequency of NPLRs across diagnostic groups was compared with that of healthy controls using Fisher’s exact test. Because the three psychiatric disorders may share underlying biological mechanisms, participants from all three clinical groups were subsequently pooled and categorized according to phase-locking status (PLR or NPLR). Phase differences and clinical characteristics between these two groups were then examined. The normality of continuous clinical variables was assessed using the Shapiro–Wilk test. Variables that violated the assumption of normality were compared using the Mann–Whitney U test. Circular statistical analyses were performed using Oriana 4 (Kovach Computing Services, UK), and all other analyses were conducted using GraphPad Prism 10.0 (GraphPad Software, CA, USA). All statistical tests were two-sided. Statistical significance was defined as p ≤0.05 for uncorrected analyses and q ≤0.05 after false discovery rate (FDR) correction using the Benjamini–Hochberg method for multiple comparisons.

## 3 Results

### 3.1 Diagnostic group comparisons

#### 3.1.1 Phase delay in the 40-Hz ASSR is a shared feature across major depressive disorder, bipolar disorder, and schizophrenia

**Figure 2:**
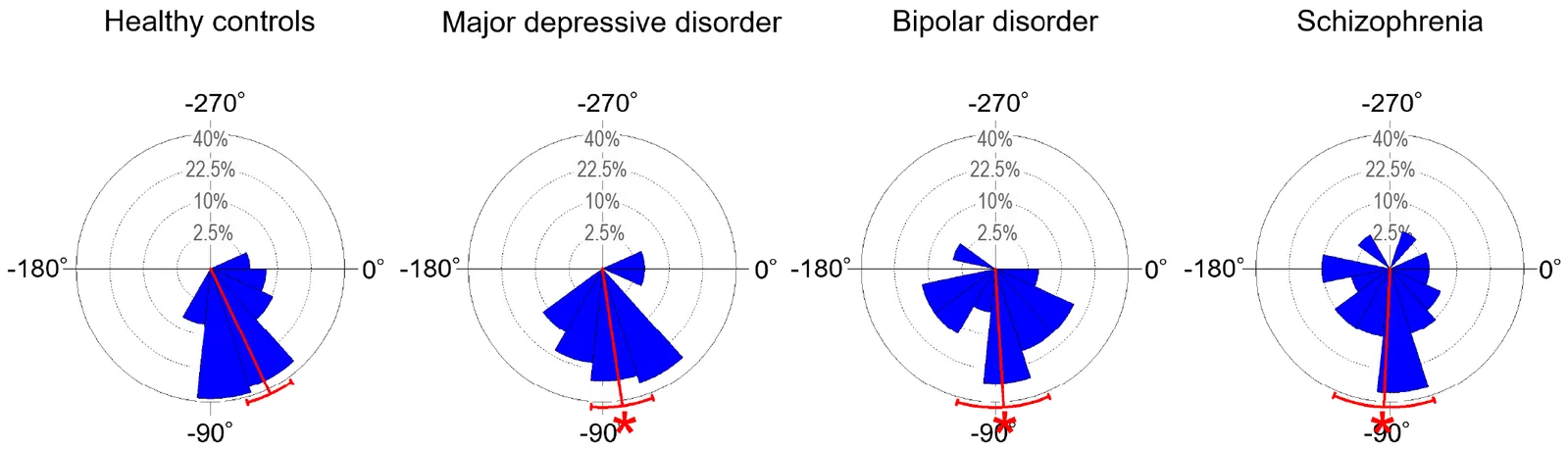
Phase delay of 40-Hz ASSR in mood and psychotic disorders. The distributions of 40-Hz ASSR phase values are shown for each diagnostic group. Blue wedges indicate the percentage of participants within each 24° phase bin, as shown on the radial scale. Compared with healthy controls, all clinical groups exhibited delayed phase responses, suggesting that slowed neural synchronization is a transdiagnostic feature shared across mood and psychotic disorders rather than a diagnostic-specific abnormality. Red lines and arcs indicate the mean phase and 95% confidence intervals. As described in **Figure 1**, phase reflects the temporal delay between auditory stimulation and neural response and is displayed as a negative angle relative to 0°. *p ≤ 0.05.

**Figure 2** illustrates the distribution of the 40-Hz ASSR phase values in participants with schizophrenia (−92.6° ± 56.2°), bipolar disorder (−86.5° ± 48.6°), major depressive disorder (−81.7° ± 33.1°), and healthy controls (−64.6° ± 28°). Compared with healthy controls, all three clinical groups exhibited significant phase delay in the 40-Hz ASSR, including participants with schizophrenia (*F*_1,56_ = 5.7, *p* = 0.021), bipolar disorder (*F*_1,51_ = 4.0, *p* = 0.050), and major depressive disorder (*F*_1,52_ = 4.1, *p* = 0.047). All comparisons remained significant following FDR correction (all q = 0.050).

#### 3.1.2 Individual-level profiling of neural synchrony detects NPLR across clinical groups

All healthy controls exhibited PLR. In contrast, NPLRs were identified in eight participants with schizophrenia, three with bipolar disorder, and one with major depressive disorder (**Table 2**). Overall, the frequency of NPLRs differed significantly among the diagnostic groups (*p* = 0.0031). Exploratory *post hoc* analyses demonstrated a significant difference between healthy controls and participants with schizophrenia (*p* = 0.0045, q = 0.014). However, the differences between healthy controls and participants with bipolar disorder (*p* = 0.086, q = 0.13) or major depressive disorder (*p* = 0.46, q = 0.46) were not statistically significant. Examination of individual clinical profiles showed that the three participants with bipolar disorder who exhibited NPLRs had relatively high BPRS total scores (19, 12, and 10). Notably, the single participant with major depressive disorder who exhibited an NPLR had prominent psychotic symptoms (BPRS = 32). Review of the subsequent clinical course revealed that this participant was the only individual in the study cohort who later underwent electroconvulsive therapy. Although based on a limited number of cases, these observations suggest that NPLRs may reflect a transdiagnostic dimension associated with greater clinical severity and psychotic symptomatology.

**Table 2:** Occurrence of phase-locked or nonphase-locked responses in 40-Hz ASSR.

40-Hz ASSR	PLR^a^ (frequency)	NPLR^b^ (frequency)	Fisher’s exact test
Schizophrenia, *n* = 29	21 (72%)	8 (28%)	*p* = 0.003^c^
Bipolar disorder, *n* = 24	21 (87%)	3 (13%)	
Major depressive disorder, *n* = 25	24 (96%)	1 (4%)	
Healthy controls, *n* = 29	29 (100%)	0 (0%)	
Total, *n* = 107	95 (89%)	12 (11%)	

^a^ Phase-locked response.

^b^ Non-phase-locked response.

^c^ Exploratory *post hoc* analyses showed a significant difference only for schizophrenia versus healthy controls (*p* = 0.005).

### 3.2 Phase-locking status (PLR vs. NPLR)

#### 3.2.1 Clinical PLR and NPLR groups show phase delay

**Figure 3:**
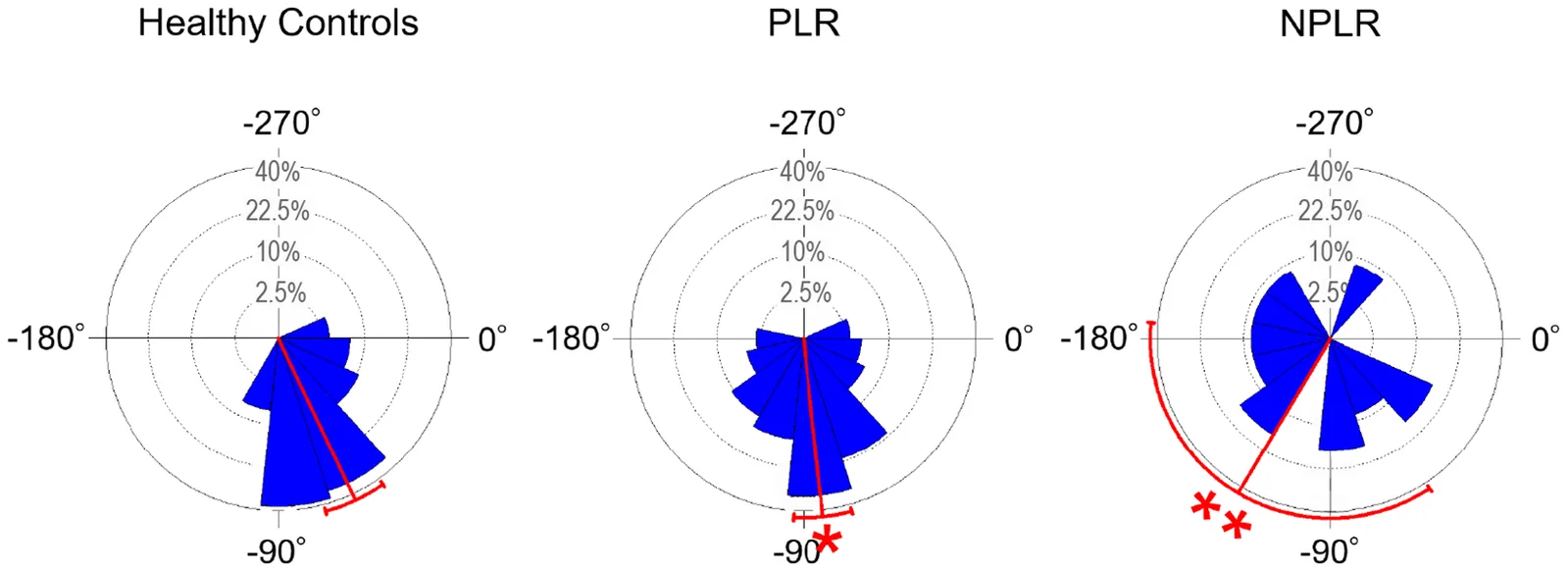
Phase delay in the clinical PLR and NPLR groups. Participants from the three clinical groups were pooled and classified as having PLR (n = 66) or NPLR (n = 12). The distributions of the 40-Hz ASSR phase values are shown for the clinical PLR, NPLR, and healthy control groups. Both clinical groups exhibited significant phase delay relative to healthy controls, with the delay being more pronounced in the NPLR group. *p ≤ 0.05, **p < 0.01.

Given the potential biological overlap among the three psychiatric disorders, all clinical participants were pooled and classified according to phase-locking status as either PLR or NPLR. Healthy controls were analyzed separately. The distribution of 40-Hz ASSR phase values for healthy controls, the PLR, and the NPLR groups is shown in **Figure 3**. The PLR group (−83.7° ± 39.8°) exhibited significant phase delay compared with healthy controls (−64.6° ± 28°; *F*_1,93_ = 5.7, *p* = 0.019, q = 0.029), whereas the NPLR group showed larger delay (−120.8° ± 78.6°; *F*_1,39_ = 7.8, *p* = 0.0082, q = 0.025). The phase delay in the NPLR group exceeded that observed in the PLR group, although this difference did not reach statistical significance (*F*_1,76_ = 3.6, *p* = 0.060, q = 0.060).

#### 3.2.2 Exploration of clinical features associated with NPLR

**Figure 4:**
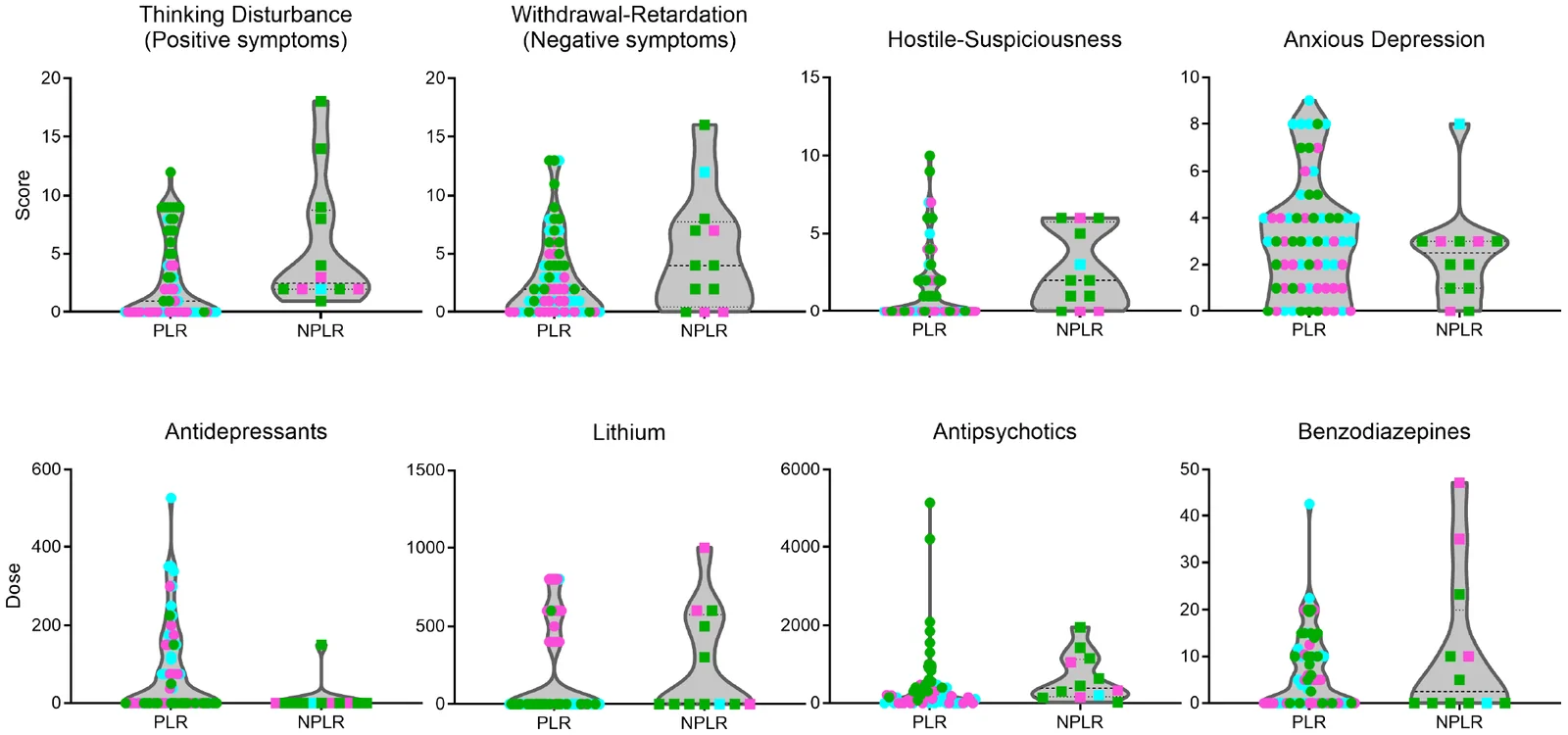
Profiling of individuals with NPLR to explore associated clinical features. Dimensional symptoms and psychotropic medication doses are shown for the pooled clinical PLR and NPLR groups. Although exploratory analyses identified nominal differences in several variables, including thought disturbance, none remained significant after FDR correction for multiple comparisons. Green, pink, and light-blue dots represent participants with schizophrenia, bipolar disorder, and major depressive disorder, respectively.

Clinical characteristics were compared between the pooled PLR and NPLR groups. No significant differences were observed between the two groups in age (t = 1.8, p = 0.081) or sex distribution (χ^2^ = 0.39, p = 0.53). Because the Shapiro–Wilk test indicated deviations from normality in at least one group for each psychiatric symptom score and psychotropic medication dose, these variables were compared using Mann–Whitney U tests. Exploratory analyses identified nominal differences between the PLR and NPLR groups in BPRS thought disturbance scores (*p* = 0.0070), antidepressant dosage (*p* = 0.017), and antipsychotic dosage (*p* = 0.025). However, none of these variables remained statistically significant after FDR correction (BPRS thought disturbance q = 0.056; antidepressants q = 0.067; antipsychotics q = 0.067). These findings are summarized in **Figure 4**.

## 4 Discussion

This study presents a practical ERA-based framework for evaluating the 40-Hz ASSR to characterize phase delay and individual-level neural dyssynchrony across mood and psychotic disorders. A novel finding is that phase delay of the 40-Hz ASSR, previously reported in schizophrenia, was also observed in bipolar and major depressive disorders. Participants experiencing overt manic episodes were excluded because EEG recordings required a stable resting state. Consequently, depressive and anxiety symptoms represented the predominant BPRS dimension in the bipolar and major depressive disorder cohorts (**Table 1**). These findings suggest that phase delay in mood disorders may be associated with depressive symptom dimensions. Recent evidence demonstrates that ketamine advances the phase of the 40-Hz ASSR in healthy individuals (33). This finding raises the possibility that the phase delay observed in mood disorders is modifiable, as the phase of the 40-Hz ASSR reflects the timing of neural responses within cortical gamma-band circuitry. Given the established clinical use of ketamine for treating depressive episodes in both disorders (34–36), delayed ASSR phase may represent a biomarker relevant to ketamine’s therapeutic mechanism or treatment response. Future studies examining changes in 40-Hz ASSR phase before and after ketamine treatment will be important for evaluating this possibility.

The NPLR identified by the ERA system appears to reflect marked disruption of neural phase synchronization, as evidenced by its occurrence in fewer than one-third of participants with schizophrenia (**Table 2**). Nevertheless, NPLRs were also detected in a small subset of participants with bipolar and major depressive disorders. The particularly low prevalence of NPLRs in major depressive disorder may help explain the inconsistent findings reported in previous ASSR studies, some of which found no significant impairment in phase locking (21, 22), whereas others reported only modest abnormalities (19, 20). In contrast, a delayed ASSR phase was observed across the major depressive disorder cohort, suggesting that phase delay may be a more sensitive marker of subtle gamma-band dysfunction than impaired phase locking. This interpretation is further supported by our finding that significant phase delay was present even among participants classified as having PLRs in the clinical groups and was more pronounced in those with NPLRs.

Exploratory analyses identified nominal associations between NPLRs and several clinical variables, including psychotic symptoms; however, none remained statistically significant after correction for multiple comparisons. Previous findings suggest that 40-Hz ASSR phase-locking deficits may be associated with psychotic features, including hallucination severity across psychotic disorders (18), and that these deficits appear to be more pronounced in individuals with psychotic features within the schizo-bipolar spectrum (17). Because NPLRs were relatively infrequent, particularly among participants with mood disorders, the present study had limited statistical power to detect reliable clinical correlates. Larger multicenter studies will therefore be necessary to clarify the clinical significance of NPLRs. Such work may help identify individuals with increased vulnerability to psychosis, while also facilitating evaluation of phase delay among participants with PLR. Ultimately, these findings could contribute to more personalized use of dissociative treatments, including ketamine, for depressive disorders.

This study has several limitations. First, the analyses pooled the schizophrenia, bipolar, and major depressive disorder to investigate shared neurophysiological features. Future investigations should also examine the clinical characteristics associated with NPLRs within each diagnostic group separately. Second, the potential influence of confounding factors, particularly psychotropic medications, was not fully controlled. Clinical characterization was also limited because detailed cognitive assessments and longitudinal measures of mood fluctuations were not available. Furthermore, a previous study has suggested that handedness may influence the 40-Hz ASSR (37). In addition, hearing status was assessed based on medical interviews and chart review, and Rinne and Weber tests were not performed; therefore, unrecognized hearing impairment cannot be completely excluded. Future research should therefore evaluate ASSR abnormalities while accounting for a broader range of demographic and clinical confounding variables. Third, ERA-based analysis is limited to frequency-domain summary measures and cannot provide more detailed time–frequency information. Finally, the ERA system does not incorporate automated removal of recording noise during ASSR acquisition. Therefore, this approach may be less suitable for individuals who are unable to remain still during the recording session and are therefore more susceptible to motion-related artifacts.

In conclusion, this study extends previous findings on ERA-based assessment of the 40-Hz ASSR by demonstrating that delayed neural phase and individual-level NPLR are detectable not only in schizophrenia but also in bipolar and major depressive disorder. These findings may support the broader application of ERA-based ASSR assessment as a practical and scalable approach for investigating gamma-band dysfunction across mood and psychotic disorders.

## Data Availability

The raw data supporting the conclusions of this article will be made available by the authors upon reasonable request, without undue reservation.
